# Organizational factors associated with readiness to implement and translate a primary care based telemedicine behavioral program to improve blood pressure control: the HTN-IMPROVE study

**DOI:** 10.1186/1748-5908-8-106

**Published:** 2013-09-08

**Authors:** Ryan J Shaw, Miriam A Kaufman, Hayden B Bosworth, Bryan J Weiner, Leah L Zullig, Shoou-Yih Daniel Lee, Jeffrey D Kravetz, Susan M Rakley, Christianne L Roumie, Michael E Bowen, Pamela S Del Monte, Eugene Z Oddone, George L Jackson

**Affiliations:** 1 Center for Health Services Research in Primary Care, Durham Veterans Affairs Medical Center, 411 West Chapel Street, suite 600, Durham, NC, USA; 2 Department of Medicine, Division of General Internal Medicine, Duke University, 2301 Erwin Road, Durham, NC, USA; 3 Department of Psychiatry and Behavioral Sciences & School of Nursing, Duke University, 307 Trent Drive, Durham, NC, USA; 4 Department of Health Policy and Management, Gillings School of Global Public Health, University of North Carolina at Chapel Hill, 135 Dauer Drive, Chapel Hill, NC, USA; 5 Department of Health Management and Policy, School of Public Health, University of Michigan, 1415 Washington Heights, Ann Arbor, MI, USA; 6 VA Connecticut Healthcare System, 950 Campbell Avenue, West Haven, CT, USA; 7 School of Medicine, Yale University, 333 Cedar Street, New Haven, CT, USA; 8 Durham VA Medical Center, 508 Fulton Street, Durham, NC, USA; 9 VA Tennessee Valley Geriatric Research Education Clinical Center (GRECC), Health Services Research & Development Targeted Research Enhancement Program for Patient Healthcare Behavior, and Clinical Research Center of Excellence (CRCoE), VA Tennessee Valley Healthcare System, 1310 24th Avenue South, Nashville, TN, USA; 10 Department of Medicine, Vanderbilt University, Nashville, TN, USA; 11 Departments of Internal Medicine, Clinical Sciences, and Pediatrics, University of Texas Southwestern Medical Center, 5323 Harry Hines Boulevard, Dallas, TX, USA

**Keywords:** Implementation, Hypertension, Blood pressure control, Organization

## Abstract

**Background:**

Hypertension is prevalent and often sub-optimally controlled; however, interventions to improve blood pressure control have had limited success.

**Objectives:**

Through implementation of an evidence-based nurse-delivered self-management phone intervention to facilitate hypertension management within large complex health systems, we sought to answer the following questions: What is the level of organizational readiness to implement the intervention? What are the specific facilitators, barriers, and contextual factors that may affect organizational readiness to change?

**Study design:**

Each intervention site from three separate Veterans Integrated Service Networks (VISNs), which represent 21 geographic regions across the US, agreed to enroll 500 participants over a year with at least 0.5 full time equivalent employees of nursing time. Our mixed methods approach used *a priori* semi-structured interviews conducted with stakeholders (n = 27) including nurses, physicians, administrators, and information technology (IT) professionals between 2010 and 2011. Researchers iteratively identified facilitators and barriers of organizational readiness to change (ORC) and implementation. Additionally, an ORC survey was conducted with the stakeholders who were (n = 102) preparing for program implementation.

**Results:**

Key ORC facilitators included stakeholder buy-in and improving hypertension. Positive organizational characteristics likely to impact ORC included: other similar programs that support buy-in, adequate staff, and alignment with the existing site environment; improved patient outcomes; is positive for the professional nurse role, and is evidence-based; understanding of the intervention; IT infrastructure and support, and utilization of existing equipment and space.

The primary ORC barrier was unclear long-term commitment of nursing. Negative organizational characteristics likely to impact ORC included: added workload, competition with existing programs, implementation length, and limited available nurse staff time; buy-in is temporary until evidence shows improved outcomes; contacting patients and the logistics of integration into existing workflow is a challenge; and inadequate staffing is problematic. Findings were complementary across quantitative and qualitative analyses.

**Conclusions:**

The model of organizational change identified key facilitators and barriers of organizational readiness to change and successful implementation. This study allows us to understand the needs and challenges of intervention implementation. Furthermore, examination of organizational facilitators and barriers to implementation of evidence-based interventions may inform dissemination in other chronic diseases.

## Background

An estimated 68 million, or 1 in 3 US adults, have hypertension [1]. The health risks of hypertension are well known and include increased risk of stroke and heart disease [2]. Controlling hypertension has been shown to reduce the risks of target end organ damage and improve cardiovascular outcomes [1]. Evidence-based mechanisms for controlling hypertension include a healthy diet, adequate exercise, and medication management [1]. However, despite the prevalence of hypertension and evidence on effective management of hypertension, it remains a serious public health problem [3,4]. This is particularly true among the US veteran population where approximately 25% to 40% of veterans with hypertension in 2007 had a blood pressure (BP) measurement ≥140/90 mmHg [5].

In an effort to address the sub-optimal BP control among veterans, the US Department of Veterans Affairs (VA) set a goal of bringing 75% of veterans with hypertension under adequate BP control, defined as less than 140/90 mmHg. To reach this goal, the VA sought to implement evidence-based interventions that would facilitate hypertension management among its patient population. As described previously [6], the Hypertension Telemedicine Nurse Implementation Project for Veterans, known as HTN-IMPROVE, was implemented in primary care practices across three Veterans Affairs Medical Centers. This evidence-based intervention has demonstrated efficacy of a nurse-delivered behavioral telephone counseling program to improve hypertension outcomes [7]. However, the intervention’s effectiveness in ‘real world’ settings, and implementation costs, remain unclear.

Our aim was to assess organizational factors associated with readiness for change to implement HTN-IMPROVE in three VA intervention sites. We sought to answer the following research questions: What is the level of organizational readiness among clinicians and other professionals to implement the intervention? What are the specific facilitators, barriers, and contextual factors that may affect organizational readiness to change?

## Methods

### Conceptual framework

**Figure 1:**
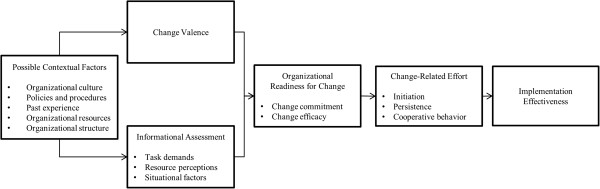
**Determinant and outcomes of organizational readiness for change **[9]**.**

We used the Weiner Organizational Theory of Implementation Effectiveness (Figure 1) [8,9], as a conceptual model to guide the identification of determinants for organizational readiness for change (ORC) [8,9]. ORC refers to organizational members’ shared resolve to implement a change and their collective ability to do so; respectively, these dimensions are known as change commitment and change efficacy. Briefly, this model posits that organizational readiness for change is the product of two constructs: change valence*,* or the degree to which organizational members value the proposed change (*e.g*., perceived need for change, perceived advantage to change, and perceived fit); and informational assessment*,* or the degree to which organizational members know what tasks are involved in the change, have enough resources available to implement the change (*e.g*., people, money and materials), and view positively situational factors such as the timing of change and the time available for implementation. Both change valence and informational assessment can be predicted by contextual factors, the broader conditions that affect an organization’s readiness for change, which may serve as both a facilitator and barrier. When organizational readiness is high, the theory posits, organizational members are more likely to initiate change, exert greater effort in support of change, and exhibit greater persistence in the face of obstacle or setbacks during implementation. The likely outcome is greater consistency and quality of intervention delivery (*i.e*., effective implementation). This theory was used as a guide for interview guide development, survey questions, and qualitative data analysis.

### Intervention overview

HTN-IMPROVE is a nurse-delivered tailored telephone intervention in which the emphasis is initiating and maintaining specific health behaviors related to hypertension. The efficacy of the intervention has previously been demonstrated through rigorous clinical trials [10-15]. The intervention is organized as telephone encounters that occur monthly for one year. At each telephone encounter, trained nurses use the intervention software to gather medical and behavioral information. This information is analyzed by the software program and activates behavioral and educational modules within the software that address hypertension related issues such as medication adherence, social support, and healthy behaviors such as limiting alcohol use, smoking cessation, weight loss, and adequate exercise [11,13,15].

The three VA intervention sites agreed to deliver the program to at least 500 individuals over a one-year period with an expectation that the patients would be contacted monthly for one year. Access to the computerized software and related technical assistance were provided by the Durham VA Medical Center Health Services Research & Development Center of Excellence staff study team. The intervention sites and the site principal investigator (PI) were responsible for ensuring the program was implemented (*e.g*., determining ways in which patient referrals occurred; working with information technology to ensure nurse interventionist needs were met). Details on implementation parameters, software operation, and patient enrollment are previously reported in the protocol [6].

### Setting

With IRB review and approval from each participating site, we implemented HTN-IMPROVE at three geographically diverse intervention sites in separate Veterans Integrated Service Networks (VISNs) which re-present three geographic regions across the US. Each participating location is affiliated with an academic medical center. Primary care is provided by attending physicians with academic appointments, non-physician providers (*i.e*., physician assistants and/or nurse practitioners), and resident physicians. The following four criteria were used to select the intervention sites: facilities perceived that could benefit from improving their level of BP control; to increase generalizability of evaluation results, demographics had to vary (*e.g*., rural versus urban, minority proportion); investigators had to have established collaboration with leaders of these VISNs; and each intervention site agreed to provide 0.5 full time equivalent employees (FTEs) of nursing time to conduct the program.

Site A includes one academically affiliated medical center and four community-based clinics. In fiscal year (FY) 2011, there were approximately 90,000 primary care encounters among 20,000 among patients. Site B includes two academically affiliated medical centers where the intervention was offered. In FY 2011, there were approximately 140,000 encounters among 61,000 patients. Finally, site C implemented HTN-IMPRVE in one academically affiliated medical center and nine community-based clinics. There were approximately 98,000 primary care encounters among 45,000 unique patients in FY 2011.

### Design

We employed a mixed-methods approach engaging both semi-structured interviews and a survey. Telephone interviews were conducted from November 2010 to August 2011, and surveys were distributed three months prior to implementation in July 2011. Participants were informed about the HTN-IMPROVE program through site visits by the researchers beginning in 2008. We conducted a needs assessment and evaluated barriers and facilitators for implementing the proposed patient-tailored hypertension self-management program at each of the three clinic sites through both semi-structured interviews and a survey. We conducted semi-structured qualitative interviews with the ‘core implementation team’ and a sample of administrators, information technology (IT) support personnel, primary care physicians, non-physician providers, and nurses, or ‘users’, who would potentially interact with the program. The ‘core implementation team’ included the intervention site PI and staff directly involved in planning and implanting the program. Exact composition varied by clinic site depending on those clinic sites’ operational needs (see Table 1). Sample interview questions included asking how committed they were to implementing HTN-IMPROVE, what they hoped to achieve by implementing the program, about other initiatives or programs underway to support implementation, and questions on resource availability. Interviews were conducted by two research assistants and were transcribed in full. Semi-structured interview methods allowed us to study implementation processes, which tend to be fluid, non-linear, and context sensitive [16,17] and allowed us to compare patterns across cases [18].

**Table 1:** Pre-implementation themes by the theory of organizational readiness for change


**Organizational situation**			
**Organizational readiness to change**			
Degree to which organization members perceive that the organization, as opposed to the individual, is prepared to implement a specific intervention.			
Positive factors		Negative factors	
+	Buy-in from administration	-	Nurse commitment hard to gauge
+	Buy-in from providers	-	Nurses may be reluctant because of other job tasks
+	Clinic accustomed to innovation	-	Clinic leaders don’t know what to expect
+	Dovetails with priorities	-	Implementation has taken a back seat
+	Clinic is committed b/c benefits are understood in terms of research, medical understanding and telehealth		
+	Core team is committed and communicating that, and wouldn't do if it wasn’t important		
**Change valence**			
Value that organizational members attribute to a proposed change.			
Positive factors			
+	Research is part of culture		
+	Self-management behavioral interventions viewed positively		
+	Increased access to care		
+	Clinicians view program will be beneficial for patients		
+	Extremely important to control BP		
**RN scope of practice**			
+	Better job satisfaction		
+	Active in patient panel		
+	Active in population management		
+	Using more skills as nurses		
+	Better job satisfaction		
**Change valence is temporary (these are both positive &negative factors)**			
+/−	Buy-in from patients needed		
+/−	Patient perspective is needed in implementation and evaluation		
+/−	Belief in program will depend on seeing evidence; thinks maybe the key is in not letting patients fall through the cracks		
+/−	Feedback from patients on satisfaction with program is important		
+/−	Interested to see if Motivational Interviewing affects HTN patient self-management		
+/−	Success depends on seeing patient data measurements (BP control; smoking; weight)		
+/−	Wait and see attitude		
+/−	Qualitative and quantitative evidence is important for continued success		
**Situational factors**			
Organizational contextual situations that affect the confidence and commitment of organizational members to implement the intervention			
Positive factors		Negative factors	
		**Timing of change effort**	
+	Aligns with clinic workflow and External Peer Review Program (EPRP)	-	Length of time to implementation is a barrier
+	Aligns with values of PACT (patient-centered care & care teams)	-	Can’t remember what HTN Improve is
+	Other programs will support HTNI (*e.g*., telehealth, MOVE, Pact, HTN clinic)	-	Don’t know what happened to HTN Improve
+	Will be better or add to current programs/patient contact frequency	-	Eager to start
**Technology**		-	Long time ago
+	Will be added to existing technology infrastructure	-	Many existing BP interventions
		-	Need to make PCP aware of program
		**Time available**	
		-	Clinic visit time is limited to introduce patients to the intervention
		-	Nurses are concerned about time available
		**Technology**	
		-	Security challenges with access to software
		-	Interoperability issues
**Task demands**			
Knowledge about the tasks that need to be performed, resources that are needed, and the time and effort that are needed to implement the intervention			
Positive factors		Negative factors	
+	Behavioral self-management intervention	-	Coordinating outreach to patient could be a burden
+	RN delivered calls	-	Implementation will be seen as adding one more thing to a nurse’s full plate
		-	Another clinical reminder
		-	Contacting patients is challenging
		-	Integration into existing workflow with minimal steps is needed
**Resource perceptions**			
Access to financial, material, or human assets to support implementation and ongoing use of the intervention			
+	Have office space	-	Need a dedicated research staff
+	Have staffing for HTNI	-	Staffing is an issue
+	Use existing equipment		
+	Have IT support		
+	Fairly knowledgeable about intervention		
**Contextual factors**			
Broader contextual conditions that affect organizational readiness for change			
+	Clinic accustomed to innovation	-	No dedicated research staff
+	Research is part of the culture	-	IT is undergoing infrastructure change
+	Past experience with implementing research		
+	Other programs will support HTNI (*e.g*., telehealth, MOVE, Pact, HTN clinic)		
+	Telemedicine is part of the VA culture and delivered by RNs		

To support our qualitative assessment and to further understand organizational readiness for change across all three intervention sites, we administered a 13-item computer-based survey, the Organizational Readiness to Change Survey (Additional file 1). This survey was based on concepts from Weiner’s Organizational Readiness to Change Theory [9]. The survey has been pilot tested and modified for use in other settings [19-21]. We selected relevant items from Dr. Weiner’s survey item data bank [9]. This survey examined perceptions of organizational-level change efficacy and commitment to the HTN-IMPROVE intervention. Survey responses were used to objectively examine ORC as a two-dimensional construct encompassing change commitment and change efficacy. We examined responses to single-item measures hypothesized to be determinants of ORC such as task knowledge, perceived resource availability, and competing priorities, among others. All responses were anonymous. The ORC survey was administered to 336 primary care physicians, non-physician providers, nurses, and information technology professionals through the VA Intranet from the three intervention sites, and a total of 102 responses were obtained (a 30% response rate).

### Qualitative analysis

Data were transcribed and then analyzed using conventional content analysis [22], a data reduction technique, to look for recurring themes in the interviews using qualitative data analysis software, ATLAS.ti 5.2 (Scientific Software Development, Berlin, Germany). Two researchers coded the data and met regularly to review coded texts, resolve discrepancies through consensus, and to discuss emerging themes. Furthermore, a third researcher analyzed a subset of the interviews to ensure reliability, agreement on emerging codes, and to help resolve any disagreements. The content analysis involved dividing interview text into segments of information and coding the segments. The conceptual model (Figure 1) provided a list of *a priori* themes, which we used to organize the emerging codes. Table 2 shows a breakdown of these codes. We examined the degree to which each code or construct appeared in the data (strength), the degree to which the construct positively or negatively affected implementation (valence), and the degree to which relationships among constructs matched the conceptual model [6]. We then examined the codes for patterns and established themes.

**Table 2:** Perceptions of organizational-level change efficacy and change commitment

	**N**	**Mean**	**Standard deviation**	**Fairly/very confident**	**Not at all/A little confident**	**Don’t know**
**Efficacy (Imp. Group)**						
Use resources effectively	71	3.28	0.70	82%	8%	10%
Encourage clinicians to try program	74	3.20	0.72	80%	14%	6%
Coordinate implementation efforts	72	3.25	0.62	82%	9%	9%
Support clinicians as they adjust	71	3.15	0.71	74%	17%	9%
Solve implementation problems	72	3.19	0.74	78%	13%	9%
				Fairly/Very Committed	Not At All/A Little Committed	Don’t Know
**Commitment (Imp. Group)**						
Committed	56	3.45	0.69	64%	8%	28%
Motivated	58	3.41	0.73	64%	10%	26%
Willing	59	3.53	0.65	69%	6%	25%
				Somewhat/Very Much	Not At All/A Little	Don’t Know
Want to	53	3.68	0.51	67%	1%	32%
				Fairly/Very Committed	Not At All/A Little Committed	Don’t Know
**Commitment (User Group)**						
Committed	61	2.84	0.80	56%	25%	19%
Motivated	61	2.80	0.87	49%	32%	19%
Willing	61	2.82	0.85	55%	26%	19%
				Somewhat/Very Much	Not At All/A Little	Don’t Know
Want to	51	3.16	0.86	56%	12%	32%
**Change Valence**				Strongly Agree/Agree	Strongly Disagree/Disagree	Don’t Know
Need for change	74	3.55	0.53	97%	1%	1%
Relative advantage	57	3.26	0.67	67%	9%	24%
Perceived fit	68	3.34	0.66	84%	7%	9%
**Informational Assessment**						
Knowledge of task requirements	56	2.91	0.82	52%	23%	25%
Resources availability	57	2.75	0.87	51%	27%	22%
Timing	57	3.02	0.79	64%	15%	21%
Competing priorities	60	2.47	0.85	32%	48%	20%
Time availability	60	2.73	0.78	51%	29%	20%
				Yes	No	Don’t Know
Presentation attendance	74	0.25	0.46	24%	73%	3%

*Note*. Commitment was assessed from two perspectives: the implementation group and the user group. The implementation group references a small, core group of people that play a leading role in implementing the program. The user group references clinicians who were expected to support the program (*e*.*g*., making referral, provide staffing).

Support for the hypothesized relationships was assessed by using three criteria proposed by Trochim [23] and Miles and Huberman [24]. First, we looked for the overall covariance of the constructs (*e.g*., whether VA intervention sites exhibiting strong implementation climate have supportive administration). Second, we looked for explicit attributions or the identification of plausible mechanisms to link the two constructs (*e.g*., participants attribute a strong implementation climate to the deployment of appropriate implementation policies and practices). Lastly, we applied the same criteria across the cases to determine if cross-case variation in implementation was consistent with the hypothesized relationships in the model.

### Quantitative analysis

Survey responses used a five-point Likert scale (*e.g*., ranging from 0 = ‘not at all confident’ or ‘don’t know’, to 4 = ‘very confident’). We performed descriptive statistical analysis to calculate percent, mean and standard deviation for the ORC Items and Scales using Stata version 10.0 (Stata Corporation, College Station, Texas) and SAS version 9.2 (SAS Institute, Inc., Cary, North Carolina). Surveys with partial responses were included.

## Results

We analyzed data from interviews (n = 27) representing three intervention sites: site A – 13 interviews; site B – 8 interviews; site C – 6 interviews. Professions/roles represented across intervention sites include: administrator – 6 interviews; physician/non-physician primary care provider – 9 interviews; nurse – 9 interviews; information technology staff – 3 interviews. Interviews averaged approximately 20 minutes. Qualitative results were summarized (Table 1) and organized according to the Weiner Organizational Theory of Implementation Effectiveness (Figure 1) [8,9].

Of the 102 respondents to the ORC survey, 74 completed it (Table 2). It is unknown why the other 28 respondents initiated but did not complete the survey. Survey responders were a mix of representatives from the three facilities. The majority of responders were clinicians, those engaged in the delivery of patient care, with limited input from non-clinical roles. Of the respondents 27 (27%) were physicians, 59 (58%) were nurses, 10 (10%) were non-physician providers (NP/PA), 4 (4%), were administrators, and 1 (1%), was an IT professional. Patterns of missing data, both in terms of non-responders and survey items, were evaluated; related to profession, intervention site, and/or survey item, there was no discernible pattern of non-response (Table 2). Below, we describe organizational attributes that lead to organizational staff members to rate a medical center’s readiness to implement a nurse-delivered telephone self-management program and specific groups of factors that would be expected to impact this readiness: situation factors, change valence, task demands, and resource availability. Results are organized according to the Weiner Organizational Theory of Implementation Effectiveness (Figure 1) [8,9].

**Figure 2:**
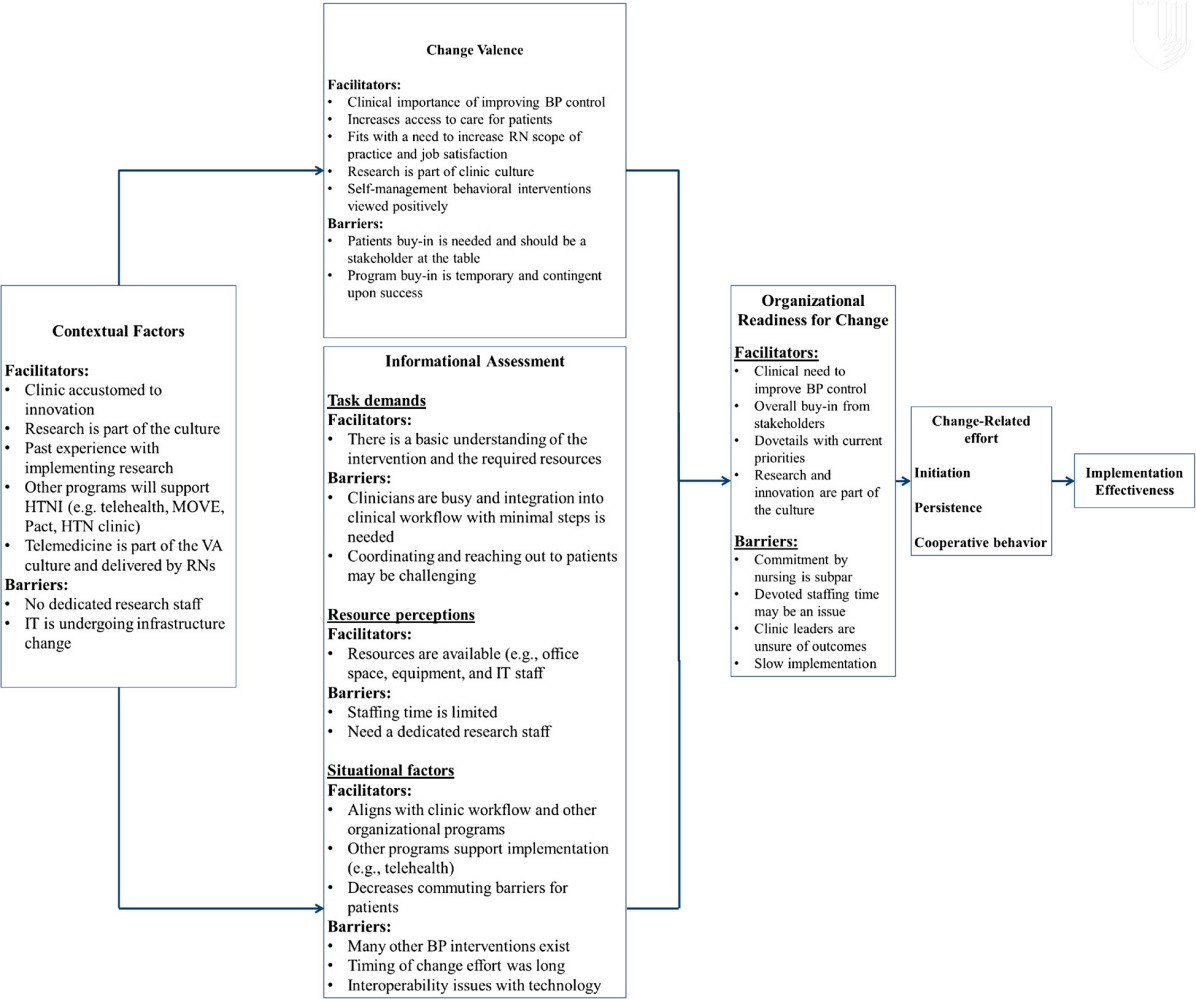
Summary of key findings.

Overall, intervention sites expressed readiness to implement the program as demonstrated by the positive results, defined as a facilitator toward implementation, from the interviews (Table 1) and survey items (Table 2). There were limited differences in readiness to implement by intervention site in the results. This included minor differences in reported resource availability. Differences in the perception of ORC did however exist between professions such that nursing was concerned with increased workload and dedicated staff time to conduct the intervention. Figure 2 presents a summary of the key findings.

### Organizational readiness to change (ORC)

ORC refers to the extent to which organizational members are prepared as a group to make the changes in organizational policies and practices that are necessary to implement and support innovation use (change commitment) and their perceived ability to do so (change efficacy) [25]. Administration was committed to implementation as demonstrated by their devotion of the required 0.5 FTE. Information technology staff members were confident in their ability to implement the software due to their previous experience with similar research and that there was buy-in from administration.

Still, the commitment by individual nurses and nursing administration was difficult to gauge. There was reluctance by the nurse interventionists due to uncertainty of time commitment and fear of creep in the scope of the time needed for the nurse-delivered telephone calls. There was also a fear that due to the length of implementation time, buy-in was waning, and the devoted nursing time would be permanently shifted to other tasks they had taken up during the pre-implementation phase. One nurse administrator said:

I would just say, with something like this, when it’s a research project, when it first comes out and the leadership is told we’re going to try this, we’re going to implement this here and then now we’re almost a year later. The timeliness of ‘we have an idea, we have a project we’d like to implement’, if it could happen within a three-month timeframe, I think buy-in and support would happen a lot better.

With regard to change commitment and change efficacy, organizational members were favorable toward the task demands required of HTN-IMPROVE, resources were adequate in the immediate future for implementation, and situational factors supported the program. Existing situational factors (*e.g*., telehealth), helped support buy-in and the view that the intervention could be implemented successfully. This is supported by responses to the survey (Table 2). Nearly 70% reported that the core group of people leading the implementation wanted to put HTN-IMPROVE into practice ‘very much’. Similarly, 69% said that the clinicians expected to support the program were either ‘fairly’ or ‘very committed’ to implement the program. Regarding motivation of the clinicians using the program, nearly one-quarter reported that they were ‘very motivated’ to implement the program, and 36% indicated that that they were ‘fairly motivated’. Of respondents, 39% reported that their clinical work group ‘very’ much wanted to implement the program, and another 43% reported that they ‘somewhat’ wanted to implement the program. Overall, the positive factors associated with implementation outweighed the negative, and the organizations expressed readiness for change.

### Change valence

Change valence refers to the value that organizational members attribute to a proposed change [9,25]. Change valence was largely positive with stakeholders expressing many benefits for patients including increased access to care, the ability to telecommute, both of which add to and complement current care. Value in the new program was expressed in large part because HTN-IMPROVE is an evidence-based intervention that has demonstrated efficacy and cost-effectiveness [7] and would fit with the organizations’ missions, goals and values of improving patient care. Ninety-three percent of survey respondents agreed or strongly agreed that ‘self-management programs fit with our approach to patient care’ (Table 2). Clinicians and administrators also perceived that the telephone-based aspect of the intervention was of particular benefit to patients; it is convenient with no need for patients to commute and would allow the intervention sites to provide cost-effective additional care to patients. One clinician stated, ‘I think the qualitative evidence should be highly stressed. You know, how are the veteran’s feeling? How are the clinicians feeling? Is there a sense of … pride in taking part in really helping the veterans out in this way and equally, if not more important, are the veterans really happy that they’re being reached out to a little more frequently?’

From a professional role perspective, nurses often expressed that the nurse-delivered intervention allowed them to practice to a fuller extent of their license and training; nurses would feel ownership of their patients, independence in their job, and increased job satisfaction. For example, HTN-IMPROVE allowed the nurses to move beyond routine clinic work and be increasingly engaged in their patients’ care, education and self-management.

### Informational assessment

#### Task demands

Task demands refer to knowledge about the tasks that need to be performed, resources that are needed, and the time and effort that are needed to implement the intervention [9,25]. Due to the length of implementation in 2011 from initial information sessions in 2008, many participants had forgotten, in part, the specific tasks involved with the program. Once the interviewer either reminded or explained the intervention, participants were comfortable with the tasks needed for intervention implementation. Knowledge of the intervention also varied by role; for example, administrators and clinicians had a good understanding of the clinical tasks that would be required to conduct the intervention. However, the IT staff was less familiar with the clinical needs and predominately focused on the IT infrastructure and programming needs of the intervention.

#### Resource perceptions

Resource availability refers to the accessibility of financial, material or human assets that can be used to support initial and ongoing use [9,25]. Overall, most participants stated that they would be able to use existing office space, equipment, and IT support to implement and sustain HTN-IMPROVE. This resonated in the survey responses (*e.g*., between 60% and 66% of respondents indicated that they generally had sufficient resources to implement the program; see Table 2). The availability and ability to use already existing infrastructure was a major positive element of the implementation. Because each institution has dedicated nursing time to devote to the project, staffing time for initial implementation is available. Thus, securing financial resources for human and material resources was less of a concern.

However, available staffing time/available individuals over the long term were a concern. Because the intervention sites had yet to implement the project, there was also concern by both the nurses and providers as to actual workload that would be added. Furthermore, due to the length of implementation time, nurses and administrators were concerned about losing the dedicated staff time to conduct the intervention. Prior to implementation, the nurse time was used for other tasks that many were afraid they would not be able to relinquish once HTN-IMPROVE went live. Administrators also noted that there were not additional financial resources available, such as a dedicated staff member for implementation.

#### Situational factors

Situational factors refer to the contextual factors that affect the confidence and commitment of organizational members to implement the intervention [9,25]. Major situational factors that arose included the following: competing demands, competing clinical programs, timing of the change effort, available time, and technological factors. Interviewees, particularly clinicians, noted that the intervention may compete with patient care needs and the limited time available per patient during a visit. This was also supported in the survey responses. Twenty percent of respondents said they did not know whether HTN-IMPROVE would divert attention for other high-priority clinical activities (Table 2). Moreover, 40% agreed or strongly agreed that the program would divert attention from other high-priority clinical activities. It may also compete with existing programs such as existing telehealth programs and the patient-centered medical home, a care setting that facilitates a team-based approach to care where the patient, family, and a variety of clinicians work together to deliver patient-centered care. For example, the rollout of HTN-IMPROVE was concurrent with the patient-centered medical home program as site B and may have been seen as a competing demand. These barriers were matched with an overall perception that there may not be enough time to implement and conduct the intervention in an already busy clinic that requires coordination between multiple players. Nurses particularly were concerned that patients might over utilize the phone contact to address other health issues and concerns that, although important, would consume substantial time that had not been allotted to the nursing staff to address. There were also staffing concerns. Even though 0.5 FTE was devoted by each intervention to conduct the intervention, clinicians were unsure who would cover the additional workload when staff are out of the office (*i.e*., sick or on vacation).

Timing of the change effort also arose as a potential barrier. Due to implementation challenges, such as IRB approval among others, significant time had lapsed since the intervention was announced in 2008 to be implemented and the time when the interviews were conducted in 2011. Staff were concerned that buy-in and eagerness to try this new intervention were waning and that staffing time devoted would be drawn away to cover other needed clinical tasks that would become non-retractable. Of the survey respondents, 21% said they did not know whether the timing was good to implement HTN-IMPROVE, while approximately 64% either agreed or strongly agreed that the timing was good (Table 2).

Despite these barriers, there were many positive factors associated with the implementation. It was noted that this would be particularly useful for patients because participation in the HTN-IMPROVE program would not be required to commute to the intervention site. HTN-IMPROVE also has many of the same values as the patient medical home and thus will complement its efforts of a group approach to patient care management. Furthermore, the intervention will be supported by other telehealth-based programs that the staff is already familiar with and perceive as valuable. Further, research was noted to be part of the intervention sites’ culture and was a facilitator of buy-in for this intervention.

Due to the large information technology component of HTN-IMPROVE, technological and situational factors were both noted as facilitators and barriers towards implementation. As anticipated, IT staff were able to speak to many of the technicalities involved with this facet of implementation. The intervention was easily added to the existing technology infrastructure and workflow. However, there were particular issues such as security and access to patient data to the software program. For example, it was unclear how covering clinicians would have quick access to the software program when the primary clinician was out of the office (*i.e*., vacation or out sick). Clinicians also explicitly spoke to an anticipated clinical reminder overload that is already rampant in the existing electronic health record [26]. Interoperability challenges were also a concern by the IT staff. Because the intervention was not implemented on a national VA level, each intervention site was concerned with how they have to fit the software program to its local IT infrastructure. Lastly, from a national organizational-level, the IT department was being restructured, and there was uncertainty as to who would be responsible for different aspects of the implementation process and ongoing maintenance of the program. This created future uncertainty in regard to resource allocation of IT staff to the project but was important for the key stakeholders to be aware of and monitor. As indicated by one of IT staff, ‘But as OI&T [Office of Information and Technology] moves further away from VHA [Veterans Health Administration], the negotiations may take longer or some things actually happen faster and some things may happen slower. It’s just… it’s a new world and we’re not sure who’s going to make the priorities’.

#### Contextual factors

Contextual factors refer to the broader conditions that affect an organization’s readiness for change [8,9]. Contextual factors were for the most part facilitators of readiness for change. The intervention sites involved in the implementation were accustomed to innovation and, in large part due to their affiliation with academic medical centers, noted that research is a part of the culture. Many of the staff, including administration, providers, nurses, and IT, had past experience with implementing experimental programs in the clinical setting. Furthermore, the staff had experience with implementing other hypertension-specific programs, including a hypertension clinic and nurse-led telehealth with hypertension elements. Lastly, telemedicine was already part of the VA culture, and staff overall expressed its perceived value. However, there were some contextual factors that had a negative influence. Though research is part of the culture, there was no noted designated research staff for the implementation of this program. Also, because of the IT nature of HTN-IMPROVE, there was concern that future support may not be available or would have to go through other channels due to the changing infrastructure of the larger VA organizations’ IT changes.

## Discussion

Organizational readiness for change (ORC) reflects the degree of commitment and efficacy among organizational members to implement a proposed change. Overall, stakeholders expressed readiness and commitment to change (Figure 2). There was buy-in across all stakeholders and agreement that BP control was valuable, and a feeling that the intervention would fit with the organizations’ missions, goals and values of improving patient care. This theme was consistent across qualitative and quantitative assessments. Resources were devoted to implement the project, IT was able to successfully configure the software to work with the existing IT infrastructure, and clinicians were ready to move forward with deployment. However, there was some hesitancy in readiness to change, principally by nursing.

These results are also similar to other VA implementation projects such as the MOVE! weight management program, which found that organizational readiness change was the most consistent factor associated with implementation [19]. However, unlike the MOVE! Weight management program, participants in HTN-IMPROVE felt there were adequate resources available to implement the intervention, and even though knowledge of HTN-IMPROVE varied by role, participants had a general understanding of the tasks involved. Even though participants may not have remembered specific task demands due to time since they attended a presentation on the program, once an interviewer refreshed their minds, they were comfortable with the intervention.

However, resources and knowledge of task demands alone are not adequate to generate efficacy. As the behavioral theorist Albert Bandura notes ‘efficacy involves a generative capability in which cognitive, social, and behavioral sub-skills must be organized into integrated courses of action’ [27]. Organizational members may perceive that the organization has the talent and resources to implement change, but may not have the confidence that the organization can mobilize these resources in a way that creates meaningful change. This confidence depends on organizational members’ belief that the conditions which they currently face are favorable for successful implementation (situational factors).

Situational factors can both raise or lower collective efficacy judgments [9,25]. Many situational factors arose including competing demands, such as the patient aligned care team or PACT. Results demonstrate that the intensity of competing demands may have a significant impact, both positive and negative, on successful implementation. Even though there were competing demands, it was noted that HTN-IMPROVE aligned closely with the goals, group approach to patient management, and values of patient-centered care with the VA’s patient-aligned care team, known as PACT [28], which may facilitate implementation. These findings were similar to the implementation of the ‘Ten Steps’ to successful breastfeeding Baby-Friendly Hospital Initiative [20], which found that situational factors such as competing demands negatively impacted the collective efficacy and ability to implement the project. However, implementation was facilitated because the program aligned with the values and goals of the facility.

### Professional role

As the interventionists and a principal stakeholder, the perception by the nurses that the nurse-delivered intervention would allow them to practice to a fuller extent of their license and training may have helped to facilitate collective efficacy of the intervention. The VA is in the process of developing protocols and policies expanding the RN role as a member of PACT teams. An organizing principle for these care teams is to utilize personnel at the highest level of their skill set. This aligns with the recommendations by the Institute of Medicine that nurses should practice to the full extent of their education and training and should be full partners, with physicians and other health care professionals, in redesigning US healthcare [29].

### Including the patient as an important stakeholder

Interviewed stakeholders expressed the importance of the intervention for the patient population and the benefits it would bring in improving patient care. However, many logistical barriers were noted as well, such as being able to successfully contact patients and the perceived utility of the intervention by patients. Nurses were largely concerned that patients would view the intervention as a time to talk about topics beyond the scope of the intervention and would use the contact number excessively. These speculations highlighted the importance that a patient be represented as an important stakeholder to include from the planning and pre-implementation phase. Perceived value and pragmatic elements could be more easily resolved and answered with this important stakeholder at the table.

### Limitations

Generalizability is limited to the organizational environment of the US Veterans Affairs Healthcare System. In retrospect, data analysis should have occurred concurrently with the interviews so that questions could be adjusted and added as needed. However, due to our own resource availability, we were limited in time commitment to be able to transcribe and analyze interviews while implementing the intervention. The response rate of the survey was around 30%, and interviews were self-selected.

Research was noted as a part of the intervention sites’ culture. The three VA intervention sites were all affiliated with research-oriented academic medical centers where clinicians and resources, particularly research, are often shared. Thus, buy-in for this translational intervention may have been easier and higher than it would be at other non-academic or research medical facilities. Furthermore, the patient was not included in the interviews and was an important stakeholder. Despite these limitations, our findings suggest many lessons learned with relevance to non-VA facilities that are moving forward with innovative changes.

### Lessons learned

1. Stakeholder engagement, available resources, a supporting environment, and perceived value are essential in establishing organizational readiness to change.

2. Main stakeholders, such as the interventionist, should be at the planning and implementation phase from the beginning.

3. The patient should be included as a stakeholder from the beginning of implementation.

4. Communication to all stakeholders should remain constant as to the developments and setbacks toward an implementation timetable.

5. Data analysis should be concurrent with conducting interviews.

6. Availability of the study staff and receptiveness to questions is valued by stakeholders.

## Conclusion

The Weiner Organizational Theory of Implementation Effectiveness [8,9] identified key facilitators and barriers of organizational readiness to change and successful implementation of HTN-IMPROVE. Results show that ORC and readiness to implement the program is primarily positive, as indicated by the perceived value of the program. However, the primary negative factors include unclear nursing buy-in, and as perceived by key stakeholders, resource availability.

There is a significant gap between discovery and delivery of evidence-based hypertension interventions [30], and health promotion researchers have focused less attention on implementation than they have on efficacy, adoption and diffusion [31,32]. This study allows us to understand the needs and challenges of intervention implementation. Furthermore, examination of organizational facilitators and barriers to implementation of evidence-based interventions may inform dissemination in other chronic diseases. Lessons learned from this project may help the VA and other health systems accelerate the translation of evidence-based medicine and implementation efforts of efficacious evidence-based hypertension programs.

## Competing interests

The authors declare that they have no competing interests.

## Authors’ contributions

GLJ and HBB were responsible for obtaining funding. RJS, MAK, and GLJ were responsible for qualitative analyses. BJW and LLZ were responsible for quantitative analyses. MAK, HBB, BJW, LLZ SDL, JDK, SMR, CLR, MEB, PSD, EZO, and GLJ contributed to the design of the study, implementation of the project, design and coordination of the study. All authors helped draft the manuscript, and read and approved the final manuscript.

## Supplementary Material

Additional file 1 Weiner’s organizational readiness to change survey items. Click here for file
