# Supplementary material for: Organizational factors associated with readiness to implement and translate a primary care based telemedicine behavioral program to improve blood pressure control: the HTN-IMPROVE study
Source: Implement Sci. 2013 Sep 8;8:106. doi: 10.1186/1748-5908-8-106 (PMC3847033; doi:10.1186/1748-5908-8-106)
Supplement: Additional file 1 — Weiner’s organizational readiness to change survey items. [file 1748-5908-8-106-S1.docx]

| **Supplemental File 1. Weiner’s Organizational Readiness to Change Survey Items** |
| --- |
| **Efficacy (Implementation Group)** |
| 1. How confident are you that the core implementation group can: |
| 1.1 Effectively use the resources that are currently available to implement the program? |
| 1.2 Encourage clinicians to try using this program (e.g., refer patients)? |
| 1.3 Effectively coordinate the efforts of those involved in implementing this program? |
| 1.4 Support clinicians as they adjust their clinical practice in response to this program? |
| 1.5 Effectively solve problems that might arise in implementing this program? |
|  |
| **Commitment (Implementation Group)** |
| 1. In your opinion, how committed is the implementation group to implementing this program? |
| 1. In your opinion, how motivated is the implementation group to implementing this program? |
| 1. In your opinion, how willing is the implementation group to work hard to implement this program? |
| 1. In your opinion, how much does the implementation group want to implement this program? |
|  |
| **Commitment (User Group)** |
| 1. In your opinion, how committed is the user group to implementing this program? |
| 1. In your opinion, how motivated is the user group to implementing this program? |
| 1. In your opinion, how willing is the user group to work hard to implement this program? |
| 1. In your opinion, how much does the user group want to implement this program? |
|  |
| **Change Valence** |
| 1. We must do something to better manage out patients’ hypertension care. |
| 1. The program described above will work better than what we are doing now to manage our patients hypertension care. |
| 1. Self-management programs fit with our approach to patient care. |
|  |
| **Informational Assessment** |
| 1. We know how to implement this program in our user group/clinic. |
| 1. We have the resources available to implement this program. |
| 1. The timing is good to implement this program. |
| 1. This program will divert our attention from other high priority clinical activities. |
| 17. We have time in our schedule to implement this program. |
| 1. Have you previously attended a presentation about this program? |
